# A unified 3D default space consciousness model combining neurological and physiological processes that underlie conscious experience

**DOI:** 10.3389/fpsyg.2015.01204

**Published:** 2015-08-27

**Authors:** Ravinder Jerath, Molly W. Crawford, Vernon A. Barnes

**Affiliations:** ^1^Augusta Women's CenterAugusta, GA, USA; ^2^Department of Pediatrics, Georgia Prevention Institute, Georgia Regents UniversityAugusta, GA, USA

**Keywords:** consciousness, 3D default space, sensory memory space, global workspace theory, integrated information theory, thalamus, corticothalamic feedback loops, meditation

## Abstract

The Global Workspace Theory and Information Integration Theory are two of the most currently accepted consciousness models; however, these models do not address many aspects of conscious experience. We compare these models to our previously proposed consciousness model in which the thalamus fills-in processed sensory information from corticothalamic feedback loops within a proposed 3D default space, resulting in the recreation of the internal and external worlds within the mind. This 3D default space is composed of all cells of the body, which communicate via gap junctions and electrical potentials to create this unified space. We use 3D illustrations to explain how both visual and non-visual sensory information may be filled-in within this dynamic space, creating a unified seamless conscious experience. This neural sensory memory space is likely generated by baseline neural oscillatory activity from the default mode network, other salient networks, brainstem, and reticular activating system.

## Introduction

The mystery of how consciousness arises is one of the biggest unanswered questions in science. Two properties of consciousness cause difficulty in elucidating its underlying mechanisms: consciousness consists of a first person experience which a third person cannot directly access and consciousness is an emergent property of a neural system which does not exist in its individual parts (Lehmann, 2013). These problematic properties of consciousness have necessitated many different hypotheses regarding how the brain works as the mind. Our model, like many other models, emphasizes the important role of corticothalamic feedback loops in consciousness and stresses the important involvement of the entire body in conscious experience. The neural aspect of our proposed consciousness model is similar to many currently accepted consciousness models, such as the Global Workspace Theory but has some key differences. We discuss evidence from physiology, anatomy, dynamic peripheral and central nervous system oscillations, homeostasis, psychology, neurophysiology, and evolution. We have recently published various aspects of this comprehensive hypothesis, including human physiology, neurophysiology, and mind-body response in theoretical articles that detail novel mechanisms that may underlie consciousness and various disorders such as contralateral neglect syndrome, motion sickness, claustrophobia, (Jerath and Crawford, 2014), phantom limb syndrome (Jerath et al., 2015b), anxiety (Jerath and Crawford, 2015a; Jerath et al., 2015a), and various other medical conditions and neural processes (Jerath and Crawford, 2015b). In this paper, we expand on our previously proposed consciousness model to include discussion on the issues of memory, internal imagery, emotions, meditation, sleep, and other topics. Our model also offers insights into various levels of consciousness such as coma, slow-wave sleep, REM and dream sleep, awake states, and higher consciousness states. Further refinement of this model has the potential to elucidate a causative mechanism that leads the self into normal and abnormal psychological states and underlies consciousness.

## Previously proposed models of consciousness

Some have proposed that sensory consciousness emerged when early mammals or mammal-like reptiles evolved larger thalamocortical networks (Tononi and Edelman, 2000). It has also been proposed that it is likely that sensory consciousness evolved long before what we think of as conscious human experience and thought. In addition, the unique experience of human consciousness likely involved the development of the ability for voluntary control of these pre-human consciousness processes (Baars, 2003). When examining consciousness in other species it is useful to use human consciousness as a reference (Edelman et al., 2005). In addition, the wealth of studies indicating that the thalamocortical system is involved in the generation of consciousness suggests that we should search for homologous structures in non-mammalian species (Edelman et al., 2005). However, this is not always the best approach. For example, studies have shown that the hippocampal formation performs similar functions in mammals and birds but differs vastly in anatomy and physiology (Striedter, 2002). It has been proposed that mammalian consciousness emerged when reentrant connectivity between brain areas evolved (Edelman, 2004). But this raises questions regarding “intelligent” invertebrates, such as cephalopods, that demonstrate learning and awareness. Reentrant connectivity, such as that exhibited by corticothalamic feedback loops in mammalian brains, has not been observed in cephalopods but some possible homologous structures have been identified (Edelman, 2004). The many studies that demonstrate the learning and behavioral abilities of octopi suggest that they exhibit sensory consciousness; however, whether this consciousness arises via a reentrant mechanism similar to that of mammalian brains or whether it arises via a different mechanism is still up for debate and requires further study. A useful distinction that Edelman makes is defining primary or sensory consciousness which consists of the creation of a neural multimodal scene and higher-order consciousness which involves the same aspects of primary consciousness, with the addition of a frame of reference that can “access” the past, present, and future, a sense of self, and the ability to construct past and future representations (Edelman, 2004). Current theories of consciousness have yet to answer what David Chalmers has termed the “hard problems” of consciousness of how the brain produces subjective experience from sensory signals (Chalmers, 1995), such as how the brain experiences what water feels like or the taste and texture of an orange. This is due to the subjective rather than objective nature of these experiences, hence the difficulty in studying and unraveling this phenomenon. Currently, there are two consciousness models that are regarded as the most current leading theories on consciousness processes: the Global Workspace Theory and the Integrated Information Theory.

Bernard Baars developed the Global Workspace Theory (GW), which he and other researchers have further developed over the years. The simplest explanation of the GW Theory is with a theater metaphor. In this model, consciousness is the bright spotlight on a stage and the surrounding dark theater is the unconscious (Baars, 2005). This model should not be confused with a “Cartesian theater” model in which the self is an entity observing what is in the spotlight; rather the self is the spotlight itself. This model involves a momentary working memory consisting of a few seconds of active and dynamic subjective experience (Baars, 1997). This working memory includes inner speech, visual imagery, and “the narrative of our lives” (Baars, 1997). According to this model, consciousness arises from interactive but independent hubs throughout the brain. These streams of unconscious parallel processing have limited interaction and “compete” for propagation. This functions by a “winner-take-all” mechanism in which multiple streams settle on a single stream for binding and propagation (Baars et al., 2013).

Rather than addressing the “hard problems” of consciousness directly, Tononi has proposed a consciousness model known as the Integrated Information Theory (Tononi, 2008) which examines consciousness itself in order to create a framework to better understand consciousness process es that give rise to the phenomenon of subjective experience. The theory consists of various basic tenants such as that conscious experience consists of the integration of large amounts of information; and this information is irreducible in complexity. According to Tononi, consciousness is integrated information. Although the integration of information is an important aspect of consciousness, consciousness should not be equated simply to integrated information. In Tononi's model, processed information from sensory systems and cognitive processes is integrated into a seamless conscious experience. In this model, the degree of irreducibility is known as *phi*. They propose that if *phi* is 0 for a system then that system is reducible, whereas *phi* must be a large number for a system to be conscious (Tononi, 2004, 2008). Tononi also proposes a “qualia space” in which the informational relationships between the various axis within this space give rise to specific experiences (Tononi, 2008).

Trehub proposes a Retinoid Model in which a retinoid system integrates separate foveal images to create a 3D space within the brain (Trehub, 2007). This retinoid system is composed of re-stimulating autaptic neurons. The separate 2D retinal images are projected on retinoid arrays to create a seamless 3D image (Trehub, 2007). He also proposes that this system also applies to the projection of non-visual information such as the representation of the body within egocentric space. This is a very important model in demonstrating how 3D visual images and non-visual representations within the mind may be produced but it does not address many issues, including the specific brain areas involved in consciousness or the extensive processing of retinal images that occurs throughout the brain. For example, at what point in visual processing does this 3D projection occur and at what point does this projection rise to conscious awareness? We propose that the projection of 3D space is processed by the thalamus after extensive processing throughout the cortex. We propose that this projection/filling-in of 3D default space from 2D retinal images may occur via a mechanism similar to Trehub's retinoid model but further studies need to be done to elucidate this and other possible neuronal mechanisms. Our consciousness model integrates important elements of these currently accepted consciousness models to create a model that addresses conscious experience and the important but overlooked roles of the thalamus, resting state networks, respiration, and the role of slow and fast oscillations from throughout the body.

Merker's proposals regarding consciousness emphasize that what we experience is more of a derivative of the actual stimuli than what is commonly thought and what we experience is an interpretation of a stimulus (Merker, 2013), which is similar to the tenets of our proposed model. Merker also proposes that nothing is added to content in order for it to rise to conscious awareness; rather, this content is formatted so that it can provide the best estimate of a stimulus within a very short amount of time. For example, in vision this content must be formatted within minute inter-saccadic intervals (Merker, 2012). In the past Merker had proposed that the upper brainstem may be the medium for conscious function (Merker, 2007) but more recently he has discussed the possible role of thalamic nuclei in implementing a “global best estimate buffer” and implementing perspective and an ego-center for interactions with the body and external world (Merker, 2012). The brainstem plays an important role in our consciousness model and we propose that oscillatory activity from the brainstem may be involved in the idling neural activity that forms the neural sensory memory space. Therefore, our model and Merker's proposals have cross-over in terms of processing of content but we have proposed a more specific and global model that further defines neural correlates, the properties and interactions of our defined neural and body spaces and further defines the framework of consciousness processing and the thalamus as the central primary hub for consciousness.

Our model shares some similarities to Damasio's model in regards to internal representations of visual and non-visual sensory information and emotions. In addition, Damasio's model proposes a proto-self which is “a coherent collection of neural patterns which constantly map, moment by moment, the state of the physical structure of the organism in its many dimensions,”(Damasio, 1999) which is similar to our concept of 3D default space. However, Damasio's model focuses primarily on emotion aspects of consciousness and does not address neural correlates beyond well-established emotion centers of the brain.

Min proposes a thalamic reticular network model of consciousness and emphasizes the role of the thalamic reticular nucleus in attention (Min, 2010). Our model proposes this as well but emphasizes the role of the entire dorsal thalamus, as well as the thalamic reticular nuclei. In addition, Min's model does not define processes or properties of consciousness beyond the proposal of the possible involvement of the thalamic reticular nucleus. We describe a more global consciousness model involving the framework of a neural sensory memory space that is filled in with processed information by the thalamus.

Although the GW Theory and Integrated Information Theory do emphasize the important and essential role of corticothalamic feedback loops they do not recognize the importance of the thalamus and instead confine the hubs of conscious experience to the cortex. The evolutionary expansion of the forebrain that has resulted in the current mammalian cerebral cortex has allowed for elaboration and increased complexity of conscious content (Merker, 2007); however, areas of the brain such as the thalamus and brainstem are essential for consciousness while the cortex is not. For example, mammals have been shown to exhibit goal seeking behavior after decortification and decorticate rats do not show any obvious impairments upon casual observation (Whishaw, 1990). Evidence has shown that children born without a cerebral cortex exhibit discriminative awareness (Shewmon et al., 1999) and in the past, large areas of the cortex have been removed in patients to treat severe epilepsy (Pendfield and Jasper, 1954); resulting in loss of information and abilities but not a loss of consciousness or “continuity of consciousness” (Merker, 2007). Though, the neocortex plays an important role in the ability to perform complex processing, the neocortex has been proposed to not be essential for consciousness (Low, 2012). Most studies examining the neural correlates of consciousness fall into one of two categories: content-based and state-based. Content–based approaches compare conscious perception of different types of content while state-based studies compare different conscious states, such as sleep and awake states (Hohwy, 2009). Both approaches have drawbacks on their own so combining the two approaches may improve consciousness studies. Many theories, including ours, combine both conscious content and states (Baars, 1997; Tononi, 2004; Jerath and Crawford, 2014). It has been proposed that these two separate approaches should be integrated into a new approach that addresses the interplay between content and state (Hohwy, 2009). We believe that our model is successful in doing this, though experimental evidence is required to further support and elucidate our model.

## 3D default space theory

**Figure 1:**
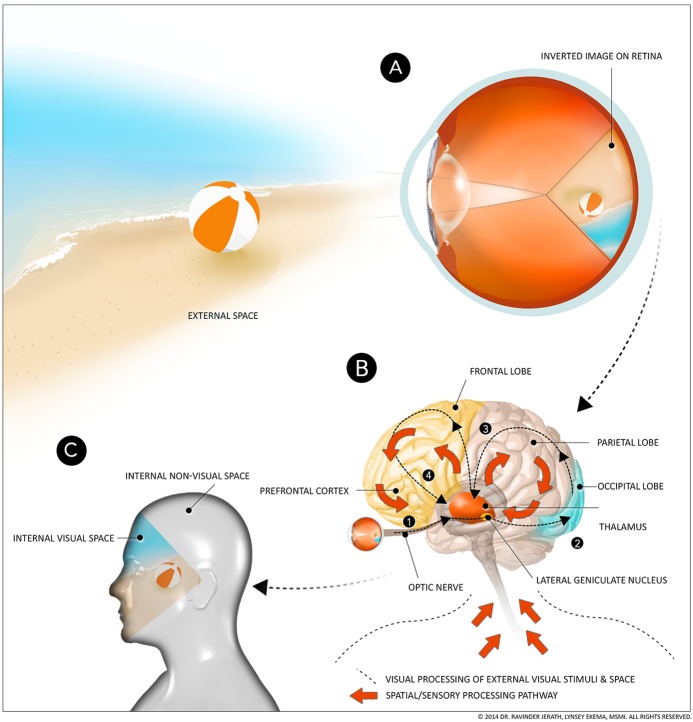
**Internal processing pathways of external visual and non-visual space**. **(A)** Projection of image on retina, **(B)** Corticothalamic feedback loops, **(C)** Visual and non-visual space. This figure depicts simplified processing pathways of visual and other sensory space. Light entering the eye forms an inverted image of the external visual space on the retina. This visual information passes through the optic nerve, through the lateral geniculate body, and other areas of the thalamus, to the occipital lobe, the parietal lobe, and then back to the thalamus. This processed information is then further processed in the frontal lobe and prefrontal cortex and back to the thalamus. This processed visual information becomes conscious when it is integrated and reimaged by the thalamus in the default 3D space. All this processing occurs in fractions of a second, so the viewer does not notice that what they are “seeing” is a processed image within their minds, rather than the actual external space. In addition to processed visual information, this default 3D space is also formed via afferent spatial/sensory input from the body. This information is involved in feedback loops between the thalamus and parietal lobe and the thalamus and frontal lobe. The thalamus integrates this processed non-visual information and forms the 3D body space within the mind. This processed information rises to conscious awareness after processing throughout the cortex and integration by the thalamus. (Figure and figure description reproduced with permission from copyright holder. Figure by Lynsey Ekema, MSMI).

Sensory information is converted into electrical signals at the sensory end organ which is then integrated and filled-in within the 3D default space. We propose that the outside world is recreated internally in a format that is optimal for interacting with and responding to the external environment. However, both attention processes and memory contribute to the final representation within and experience of conscious events. Processed external and internal stimuli are recreated within this dynamic and active sensory memory space in which current conscious experience, dreaming, and recollection of memories takes place. Our consciousness model includes components from the GW theory such as the important role of corticothalamic processing. However, in our model, corticothalamic feedback loops process sensory information from throughout the body and this information is then integrated by the thalamus and filled in to the 3D default space (Jerath and Crawford, 2014) (Figure 1).

**Figure 2:**
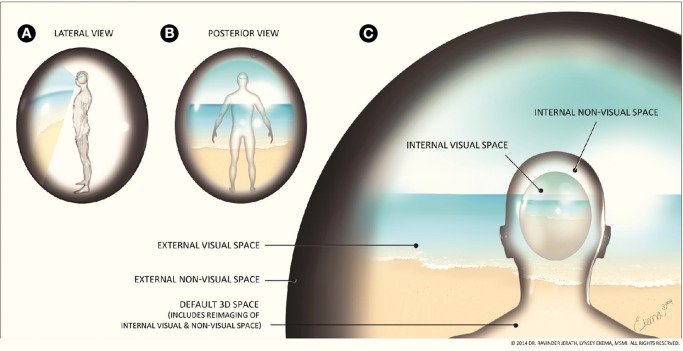
**Correlation of external and internal space**. **(A)** Lateral view of 3D default space, **(B)** Posterior view of 3D default space, **(C)** 3D default space. An important aspect of consciousness is the processing of sensory information. This figure aims to show the processing of visual sensory information and the default 3D space created in the mind. The area surrounding the figure represents the external visual space and the black surrounding area represents external non-visual space. The global visual field is surrounded by this dark shading to illustrate where the figure's visual field ends. This external visual space is seen by the eyes, processed by the brain, and an internal visual representation of external visual space is imaged within the mind, along with non-visual space. We refer to this reproduction of visual and non-visual space as the 3D default space. Note that this representation of the internal representation of external visual space illustrates this phenomenon and is not meant to indicate any anatomical landmarks in the brain. The outline of the figure is also surrounded by dark shading to show where internal space ends and reflects how the external non-visible space is mirrored functionally within the internal non-visible space. The brain is constantly receiving internal information from throughout the body and external information via the sensory systems that contribute to the formation of consciousness, a sense of self, and a 3D default space consisting of integrated and reprojected internal and external space The external visual and non-visual world appears separate from our internal world due to the high speed processing of corticothalamic information. This illusion allows the brain to function as the mind in a seemingly seamless reality. (Figure and figure description reproduced with permission from copyright holder. Figure by Lynsey Ekema, MSMI).

We propose that conscious experience arises when this space is filled in by input from the thalamus, as opposed to the GW theory in which consciousness can arise anywhere throughout the cortex. This 3D default space is home to a neural global workplace in which external space is represented internally (Figure 2). The human body consists of intrapersonal space that is composed of all the interconnected cells of the body, which are all connected and communicate via gap junctions and electrical potentials (Saez et al., 2003); we have termed this the 3D default space. It is only when the thalamus receives processed sensory information from the cortex that it integrates and fills in this information within this space. We propose that the sensory memory space (a neural component of 3D default space) may be formed by oscillations from the default mode network, resting state networks, reticular activating system, and possibly other networks. The thalamus (including the reticular thalamic nucleus) fills in this space with visual and non-visual representations using integrated and processed sensory information. This step is essential for consciousness to emerge. Therefore, the filling-in of this space and the emergence of consciousness occur simultaneously but the neural space or framework, which is filled in with processed information, is already formed by “idling” neural oscillatory activity. 3D default space consists of the entire brain and body and all the communicating cells within. This 3D default space also includes a neural component consisting of a neural sensory memory space which is filled in by both visual and non-visual processed sensory information. We propose that thalamus acts as an internal engine of awareness and is the primary hub for consciousness, though obviously many areas are involved and act as secondary or tertiary hubs. The thalamus coordinates, integrates, and orients sensory information in order for it to rise to conscious awareness.

All sensations seem to arise from outside our mind but this is an illusion inherent in the processing in the mind that helps us to quickly and efficiently interact with the outside world. For example, vision is perceived at the end organ and appears external to ourselves but what we actually perceive is processed visual information that has been recreated within the mind. In our model, we emphasize the important role of the mind-body connection in consciousness. Conscious experience arises in the mind from processed information from throughout the body and feedback from the body influences neural activity.

The thalamus is involved in attention and processing of interoceptive and self-referential activity, in addition to exteroceptive activity (Farb et al., 2013) therefore, the removal of the thalamus would likely result in loss of consciousness. Following thalamotomy, an invasive procedure performed to treat tremors in Parkinson's patients, only small portion of the thalamus is destroyed but this can lead to complications in vision and speech (Bruce et al., 2004), especially following bilateral procedures. Following thalamic infarct, distinct syndromes and dysfunctions arise dependent on the thalamic areas affected (Del Mar Sáez De Ocariz et al., 1996). For example, patients can experience memory impairment, sensory motor-dysfunctions, speech disturbances, amnesia, and many other cognitive and/or consciousness disorders. Compared to infarcts in other thalamic areas, hemorrhages, and paramedian thalamic infarcts lead to altered states of consciousness including coma, periods of stupor (Del Mar Sáez De Ocariz et al., 1996), or decreased arousal (Schmahmann, 2003). In addition, central thalamic deep brain stimulation may be used to restore consciousness and cognitive function in coma and vegetative state patients; however, further study of this as a possible successful treatment is needed (Shah and Schiff, 2010). There is a lack of literature regarding the experimental removal of the entire thalami therefore, the effects of such a procedure can only be inferred; however, because certain thalamic lesions can cause unconsciousness, removal of the entire thalamus would also result in a loss of consciousness. Bilateral thalamic tumors are rare but when they occur surgical removal of the tumor is considered inoperable (Di Rocco and Iannelli, 2002). Surgery is performed only to perform a biopsy on the tumor and prognosis is poor with only 7.6% of patients surviving more than 12 months (Gelabert-González et al., 2007). Symptoms include high incidences of mental impairment, increased intracranial pressure (Gelabert-González et al., 2007), tremors (Di Rocco and Iannelli, 2002), memory, motor, and sensory dysfunction, personality changes (Rajput et al., 2010), and other dysfunctions depending on the thalamic nuclei affected. Even bithalamic gliomas, which are generally benign, have a poor prognosis due to the involvement of thalamic nuclei and very limited surgical options (Rajput et al., 2010). Attempts to remove even small portions of these tumors can lead to severe complications including unconsciousness, coma, and overall deterioration (Rajput et al., 2010). Injury to the thalamus is associated with severe impairment of functional integration, regulation of arousal (Schiff, 2008), and deficits in executive function, memory, and attention (Van Der Werf et al., 2003). Unilateral lesions of the thalamus can result in hemispatial unawareness (Heilman et al., 1987) and altered states of consciousness such as manic delirium (Bogousslavsky et al., 1988). Thalamic infarct can also lead to widespread decrease in cerebral metabolism of the ipsilateral hemisphere (Szelies et al., 1991) due to synaptic downregulation of distant neurons connected to the thalamus (Nguyen and Botez, 1998). Lesions in the anterior thalamic nuclei cause severe spatial deficits and disrupt various types of spatial and nonspatial learning, indicating the important role of the anterior thalamic nucleus in spatial and memory processing systems (Aggleton and Nelson, 2014). Focal injuries to the thalamus can also lead to epileptic seizures (Van Domburg et al., 1996) and exhibit similar symptoms to catatonia and dystonia (Schiff, 2005). It has also been found that the loss of the integrity of the thalamus in traumatic brain injuries underlies subsequent dysfunction in executive function, memory, and attention (Little et al., 2010). In the same study, reductions in fractional anisotropy in corticocortical and corpus collosum areas of the brain did not account for significant reductions or impairment in neurophysiological function (Little et al., 2010). This study demonstrates the important role of the thalamus in executive function when compared to the role of cortex and corpus collosum. The thalamus has also been found to be an essential component of emotion and cognitive processing (Wojtecki et al., 2014). A large study on disorders of consciousness examined the links between thalamic mechanisms with willful behavior and found that levels of arousal and wakefulness were associated with atrophy of the thalamus and basal ganglia (Lutkenhoff et al., 2015).

It is widely known that the thalamus functions as a relay of sensory information to the cortex but other thalamic roles are not as well-understood. For example, the thalamus is also involved in basic corticocortical communication. The lateral geniculate nucleus acts as a first order relay while nuclei such as the pulvinar and medial dorsal nucleus act as a higher order relays that transmit information from layer 5 of the cortex to other cortical areas (Sherman, 2005). In addition, overall there are significantly more higher order relay nuclei than first order relays (Sherman and Guillery, 2001). These studies demonstrate the central role of the thalamus in cortical functioning and the flow of information. It has even been proposed that corticocortical connections may act as modulators while the majority of information is sent between cortical areas by higher order thalamic relays (Sherman, 2005). In addition, thalamic neurons are physiologically and anatomically specialized to support and maintain widespread network activity and neuronal firing patterns within corticocortical networks and thalamocortical feedback loops (Schiff, 2008).

Some researchers may argue that olfactory sensory processing goes against a thalamus-centric consciousness model since the olfactory system is the only sensory system that bypasses the thalamus (Kay and Sherman, 2007) but this is not the case. Olfactory sensory information is instead relayed to the cortex via the olfactory bulb, a structure that is very similar to the thalamus in both structure and function (Kay and Sherman, 2007). The thalamus receives the majority of olfactory sensory information indirectly via the cortex (Price and Slotnick, 1983; Price, 1985), with a few fibers connecting the olfactory bulb to the mediodorsal thalamus (Öngür and Price, 2000). Although received indirectly, olfactory sensory information is received and integrated by the thalamus similar to all other sensory systems. Odor stimulants have been shown to induce corresponding beta oscillations in both the olfactory cortex and the mediodorsal thalamus and the activity of these cortical and thalamic areas is “tightly related” (Courtiol and Wilson, 2013). In addition, many animal and human studies have indicated that the mediodorsal thalamus plays a role in odor discrimination and olfactory learning (Tham et al., 2009). The importance of the thalamus in olfactory processing and perception can also be seen in patients with thalamic lesions. These patients have been shown to have significantly impaired olfactory abilities, demonstrating the important role of the thalamus in olfaction (Sela et al., 2009).

The important role of corticothalamic feedback loops in consciousness is well-supported and many consciousness models involve information being sent back and forth between the thalamus and cortex (Edelman, 2004; Baars et al., 2013). There are also many models and studies that demonstrate the important role of the thalamus in consciousness (Min, 2010). The thalamus used to be thought of as simply a relay station for sensory information but recent studies have shown that the thalamus is actually involved in sensory processing (Cudeiro and Sillito, 2006; Ward, 2013). Sensory-specific thalamic structures are likely involved in multi-sensory integration (Tyll et al., 2011). In addition, the thalamus, in coordination with the brainstem, regulates levels of consciousness and alters levels of consciousness during sleep (Steriade, 1992). It has been suggested that there is a central hub of consciousness that synchronizes neural activity into conscious content and the role of the thalamus in relaying and receiving feedback from the cortex makes the thalamus a prime candidate for this hub of consciousness (Min, 2010).

In our model we propose that conscious experience arises when the dynamic neural sensory memory space is filled in by input from the thalamus. Many studies discuss this phenomenon of the filling-in of neural representations in consciousness (Pollen, 2003). In addition, studies on afterimages suggest that positive afterimages are likely the result of representations filled in by cortical activity (Holcombe et al., 2004) and perceptual filling in occurs in the blind spot on the retina where there are no photoreceptors (Spillmann et al., 2006). The mechanisms underlying both neural representations and filling-in within these representations are highly debated (Spillmann et al., 2006). We propose that the important role of the thalamus in sensory processing, integration, and consciousness suggests that one role of the thalamus may underlie the filling in of neural representations of both visual and non-visual sensory information.

Our consciousness model focuses predominantly on sensory consciousness and integration because these are some of the most widely researched and accessible areas of consciousness. Human subjects are able to describe their sensory experiences under various conditions and we can observe the responses of animal subjects to sensory stimuli. Interpretation of this data in combination with neural electrical activity, neural blood flow, etc., gives insights into sensory consciousness processes. In addition, we believe that sensory integration and direction of attention, which occur via the thalamus, are key steps for conscious perception of a stimulus and consciousness to occur; therefore, our consciousness model emphasizes the role of sensory integration. Sensory integration may combine processed sensory information into a format which allows for conscious awareness. Of course, sensory integration is a process and information is sent back and forth between the thalamus and various cortical areas for further processing and integration; therefore, it is unclear at which point this information arises to consciousness. The specifics of this model, beyond what we have described, require further study to elucidate. We propose that thalamocortical oscillations resonate with incoming sensory information and corticothalamic processing, integration, and attention processes all contribute to formatting information so that it rises to conscious awareness. The specifics of the frequencies of oscillatory activity that may access conscious content require further investigation; however, many studies have found widespread phase synchronization of gamma oscillations is associated with conscious perception (Melloni et al., 2007; Doesburg et al., 2009). Electrical stimulation of the thalamic reticular nucleus induces gamma oscillations in the cortex (Macdonald et al., 1998) and widespread gamma oscillatory activity has been shown to originate in the thalamus (Ribary et al., 1991). During the induction of slow-wave sleep the thalamus and cortex disconnect and deactivation of the thalamus has been shown to precede cortical deactivation by several minutes (Magnin et al., 2010). Slow-wave sleep is dominated by globally coherent slow oscillations (Steriade et al., 1993); however, these slow oscillations may have the ability to trigger faster oscillatory activity such as sleep spindles and beta and gamma oscillations (Steriade et al., 1996). These slow oscillations and gamma activity are coupled (Mena-Segovia et al., 2008) but this gamma activity is local rather than the widespread thalamacortical activity of waking states (Valderrama et al., 2012). These studies suggest that widespread corticothalamic gamma oscillations, originating from the thalamus, may underlie consciousness. Furthermore, attentional gating by the thalamus, particularly the thalamic reticular nucleus (TRN), is likely essential for what processed information rises to conscious awareness and which information remains unconscious.

Many studies have indicated that the thalamic reticular nucleus (TRN) likely plays an important role in attentional gating (McAlonan et al., 2000; Lam and Sherman, 2011) and sensory processing and integration (Crabtree, 1999). Francis Crick proposed that the TRN controls an “internal attentional searchlight” which directs attention within the brain (Crick, 1984). He and others have proposed that this attentional searchlight occurred via the rapid burst firing of the TRN (Crick, 1984). A study on classical conditioning showed the TRN is likely involved in attentional gating (McAlonan et al., 2000). Another study found that some somatosensory TRN neurons receive input from multiple thalamic regions and nuclei, suggesting that some TRN neurons integrate multiple thalamic inputs and may direct attention by inhibiting or disinhibiting certain thalamic relay cells and effectively gating sensory information sent to the cortex (Lam and Sherman, 2011). The TRN has been hypothesized to be involved in regulating cortical oscillations and regulating waking and sleeping states. For example, selective drive of TRN causes thalamic burst firing and cortical spindles from tonic firing (Halassa et al., 2011). Sensory projections from the TRN regulate sensory processing and limbic projections from the TRN are involved in regulating arousal. The TRN's involvement in regulating both sensory and limbic processing demonstrates the TRN's role in regulating both processing of both external and internal information (Halassa et al., 2014) and suggests the TRN's major role in consciousness. In fact, some previous researchers have proposed a RTN-centric model of consciousness in which a conscious state consists of TRN-modulated corticothalamic synchronization (Min, 2010).

Attention and consciousness are highly related and some researchers even equate the two but these processes should not be considered one and the same (Koch and Tsuchiya, 2007). This can be illustrated by the fact that subjects can be aware of an object without input from top-down attention processes and subjects can attend to objects they are not consciously aware of (Koch and Tsuchiya, 2007). Both the Global Workspace Theory and our model emphasize the important role of working memory in consciousness. Memory and attention both play important roles in constructing moment to moment experiences within the active sensory memory space. Attention processes and memory input are integrated with processed incoming sensory information to fill in the sensory memory space. Although conscious experience appears to be seamless, many studies have shown that information processing is likely done in distinct snapshots, similar to a movie reel that is made up of separate images (Marchetti, 2014). Marchetti points out that we can both relive past events and envision future events and that actually perceiving an object “feels” different than imagining that same object, which feels different than remembering that object. However, all of these consciousness processes involve the active construction of these events using both attention and working memory (Marchetti, 2014). We propose that all these processes occur in the neural sensory memory space of the 3D default space and that even the filling-in of temporal conscious events by the thalamus is influenced by attention and memory.

Due to the fundamental importance of the function of consciousness in regards to consciousness studies and models we should discuss the view promoted by our model. Our view on the function of consciousness is that it allows for optimal interactions with the environment and it is involved in decision-making. In a paper on the function of consciousness, Earl proposes that many actions are the result of unconscious automatic response programs while conscious experiences such as thoughts, decision-making, planning, and non-automatic responses involve a flexible response mechanism (Earl, 2014). Much of the processing done by the brain is unconscious and the ability of content to rise to conscious awareness allows for flexible responses in situations where an automatic response may have resulted in sub-optimal or negative consequences. This is evidenced by the loss of feelings or emotions being associated with poor decision-making. For example, patients with damage to areas of the brain involved in generating emotions exhibit dysfunctional decision-making and reasoning (Damasio, 1995). In addition, patients with blindsight do not make intentional responses to visual stimuli in their blind hemifields even though they have unconscious knowledge of the stimuli (Persaud and Cowey, 2008).

Due to the role of thalamus in relaying, processing, and integrating both sensory information and intrinsic conscious content, it is inherently difficult to test whether the thalamus is a primary hub for consciousness. However, it is inherently difficult to test any consciousness model, due to the subjective nature of conscious experience and the emergent properties of consciousness from neural activity as a whole rather than individual parts (Lehmann, 2013). We can, however, examine and test specific predictions made by consciousness models. Due to the vast thalamocortical feedback loops involved in consciousness, it is difficult to pinpoint whether consciousness arises in the cortex or thalamus because the thalamus supplies information to the cortex for processing and this information is feedback and forth. In previous papers, we examined disorders such as contralateral neglect syndrome (Jerath and Crawford, 2014) and phantom limb (Jerath et al., 2015b) to gain insights into consciousness and sensory processing; however, even in the examining these disorders it is difficult to determine which structures give rise to consciousness. For example, in contralateral neglect syndrome, lesions in the right parietal lobe lead to neglect of the left visual scene and left side of the body. We proposed that sensory information cannot be spatially located in the damaged parietal lobe and this damaged or lost information cannot rise to conscious awareness when it is integrated with other sensory information and filled-in by the thalamus (Jerath and Crawford, 2014). However, according to the Global Workspace Model, one might propose that the sensory information from the left side doesn't rise to conscious awareness because it cannot rise to conscious awareness within the damaged parietal lobe (or be further processed and rise to conscious awareness elsewhere in the cortex) and not the thalamus. At this point, we must rely on indirect evidence to provide clues as to the underlying mechanisms of consciousness and make testable predictions from our models.

Our model proposes that the thalamus is the central hub involved in multisensory integration and the majority of sensory processing and integration is unconscious. It is not until this information is processed and integrated into a format that can arise to conscious awareness, such as in the form of gamma oscillations that it becomes consciousness. Further, research is needed to elucidate this model and the specific frequencies associated with consciousness and various aspects of unconscious processing. The Global Workspace Model proposes that consciousness is necessary for multisensory integration (Baars, 2002); however, studies have shown that consciousness is not necessary for integration (Mudrik et al., 2014) and multisensory integration can occur in complete unawareness (Faivre et al., 2014). Though, unconscious integration has limitations and consciousness likely enables the establishment of integration mechanisms which can then operate unconsciously, as well as wider-range integration (Mudrik et al., 2014). These studies highlight the important relationship between integration and consciousness but they also illustrate that consciousness is not required for integration as proposed by other models such as the Global Workspace Model and consciousness cannot be equated to integrated information such as in the Integrated Information Theory. Mudrik and colleagues found that consciousness is necessary for long range integration but not short range integration of spatiotemporal information (Mudrik et al., 2014) which may support our proposal that gamma oscillations may be the frequency involved in conscious awareness. Widespread gamma synchrony is associated with consciousness perception (Melloni et al., 2007); therefore, this oscillatory activity may be associated with the long range integration involved in consciousness. In addition, consciousness is necessary for integration of novel information but is not required for previously learned integration (Mudrik et al., 2014).

The Global Workspace Model also proposes that in the absence of awareness there is no feedback from the frontal areas of the brain (Baars, 2005). In experiments on conscious and unconscious stimuli, masked words resulted in drastically reduced activation throughout the brain, when compared to unmasked words, and were undetectable in prefrontal and parietal areas (Dehaene et al., 2001). This study and studies that show increased metabolism in frontoparietal areas during restful wakefulness (Raichle and Mintun, 2006) suggest that frontoparietal networks play an important role in consciousness (Tagliazucchi et al., 2013a). In addition, hypometabolism in frontoparietal areas has been implicated in unconscious states such as deep sleep, coma, anesthesia, loss of consciousness during epileptic episodes which is also consistent with the Global Workspace Model (Baars, 2005). However, it has not been established whether sleep is associated with a breakdown in global connectivity or disconnection of the frontoparietal area alone (Tagliazucchi et al., 2013a). In addition, a masking study found that prefrontal networks, including the inferior frontal cortex, can be activated during unconscious trials (Van Gaal et al., 2010), demonstrating prefrontal activation from unconscious stimuli. We propose that frontoparietal networks play an important role in consciousness but that frontoparietal activation is not the “linchpin” of consciousness, rather processed information from frontoparietal areas must return to the thalamus to rise to consciousness.

Our 3D default space model proposes that the default mode network (DMN), other resting state networks, reticular activating system, and cardiorespiratory oscillations create the idling neural activity of the brain on which faster oscillatory activity builds and that forms the neural sensory memory space that is filled in by the thalamus. Low frequency fluctuations of the DMN correlate with respiratory oscillations; however, the effects of respiratory and cardiac oscillations on DMN analysis is poorly understood (Birn et al., 2008). As stated earlier, these respiratory and hemodynamic fluctuations are generally considered artifacts; however, Yuan and colleagues found significant correlation between alpha, respiratory, and BOLD signals, and suggested that this oscillatory activity may have a common neuronal origin (Yuan et al., 2012). Though few studies have examined the role of cardiorespiratory oscillations in DMN activity, many studies have established that the DMN plays an important role in consciousness. For example, unconsciousness due to epileptic seizures may be due to inhibition of brainstem arousal systems that maintain the DMN during awake states (Danielson et al., 2011). In addition, levels of DMN connectivity correlate with behavioral signs of awareness and levels of consciousness in vegetative, minimally conscious (Fernández-Espejo et al., 2012), and brain-damaged patients (Vanhaudenhuyse et al., 2010). Deep sleep has been shown to disrupt frontoparietal DMN activity but not inactivate it completely (Tagliazucchi et al., 2013b). This raises the question: if DMN activity enables consciousness then why is the DMN still active during an unconsciousness state such as deep sleep? (Tagliazucchi et al., 2013a) It has been proposed that this preserved DMN connectivity may be due to homeostatic processes and baseline neural activity of the brain that persists even in deep sleep (Raichle, 2010), which is consistent with our hypothesis.

## Testable predictions of our model:

- Thalamus is the central hub of consciousness that is highly involved in integrating and processing sensory information and allows for consciousness to arise from processed information from the cortex.
- Multisensory integration is primarily an unconscious process, as opposed to other models that propose consciousness is needed for multisensory integration.
- As other consciousness models have proposed, frontoparietal networks are important for consciousness; however, we propose that information processed by these networks must be sent back and integrated by the thalamus to rise to conscious awareness.
- Thalamic and corticothalamic gamma oscillatory activity likely underlies consciousness and which processed information rises to conscious awareness.
- First layer of neural activity consists of default mode network, resting networks, reticular activating system, and cardiorespiratory oscillations create baseline neural activity on which other activity builds and these oscillations create the neural sensory memory space that is filled in by the thalamus. This layer consists of primarily slow oscillations and delta frequencies.
- Second layer of neural activity consists of limbic activity which may be composed primarily of beta and theta oscillatory activity.
- Third layer consists of corticothalamic activity and higher processing which is composed primarily of alpha and gamma frequencies.
- The second and third layers of neural activity build on the slow oscillatory activity of the first layer and are involved in processing information that the thalamus fills into the active sensory memory space.

## Testing consciousness models

The central deep location of the thalamus within the brain, as well as the important functional role of the thalamus in many neural processes makes it difficult to study in terms of consciousness. Complete removal of the thalamus would likely result in coma or death therefore, it is difficult to determine the possible role of the thalamus as the central hub for consciousness, as we have proposed in our model. Various studies have inactivated areas of the thalamus but inactivation of the entire thalamus has proven difficult. For example, injection of muscimol, a GABA_A_ receptor agonist, or tetrodotoxin into the somatosensory thalamus inactivates this area of the thalamus and results in enhanced slow membrane potential fluctuations in cortical neurons during quiet wakefulness but hyperpolarized and suppressed these fluctuations during active wakefulness (Poulet et al., 2012). Inactivation of this thalamic area resulted in a membrane potential variance of close to zero in corresponding cortical neurons (Poulet et al., 2012). These studies demonstrate thalamic control of cortical activity and give methods of inactivating areas of the thalamus which could be applied to studying consciousness models. In addition, expression of channelrhodopsin-2 in the somatosensory thalamus and exposure of the area to blue light via optical fibers is another method that has been used to activate and inactivate the somatosensory thalamus (Poulet et al., 2012). The enhancement of slow-waves by inactivation of the somatosensory thalamus may be similar to the enhancement of slow-waves that occurs during non-REM sleep, when the thalamus and cortex deactivate (Magnin et al., 2010), though on a much smaller scale and during an awake state. Injections of muscimol, tetrodotoxin, or optigenetic deactivation are various methods that could be attempted on a larger scale and applied to the entire thalamus to examine the possible role of the thalamus as the central hub of consciousness. However, the thalamus and cortex are so inextricably linked that the thalamus is likely required for consciousness that may arise via various cortical hubs. Therefore, loss of consciousness due to complete removal of the thalamus or inactivation of the thalamus does not preclude the cortex from being a consciousness hub. To determine whether consciousness hubs arise within the cortex or via the thalamus we would have to interfere with the reentrant feedback loops of corticothalamic processing at various feedforward and feedback processing stages. Considering the millisecond-timescale of corticothalamic processing and the vast parallel processing that occurs throughout the cortex, this presents seemingly insurmountable obstacles for current technology. Currently available technology necessitates focusing on smaller-scale studies, such as the experiments discussed above, to provide insights into how and where consciousness arises. Injection of muscimol or tetrodotoxin is not likely feasible to perform on a millisecond time scale; however, optigenetic deactivation may possibly be feasible on the needed timescale. For example, optigenetic deactivation of the lateral geniculate nucleus in the thalamus could be performed to study visual consciousness or deactivation of the medial geniculate nucleus could be performed to study auditory consciousness. Optigenetic deactivation of the appropriate thalamic nuclei could be performed starting at various millisecond increments after exposure to a stimulus. This stimulus would need to elicit a known or established evoked-potential response within the brain of the animal model being used. Judging the point of conscious awareness as the point at which a behavioral response to the stimulus was observed from the animal would likely not give an accurate moment of conscious awareness. This type of study should be followed by optigenetic deactivation of the corresponding cortical sensory area, following the same timeline. The point at which the known evoked-potential response is elicited in both the thalamic and cortical experiments could give insights as to the point at which the stimulus reached conscious awareness and reveal whether consciousness arises in thalamic or cortical hubs. Further research is needed to foster better experiments to study consciousness models such as the 3D default space model we have proposed.

## Conditions and phenomena explained by our model

**Figure 3:**
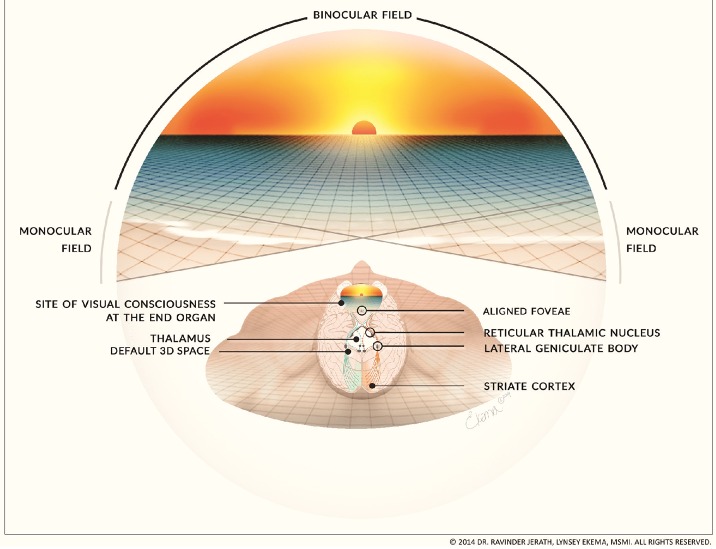
**Intrinsic spatial mapping of visual and non-visual stimuli within the 3D default space**. This figure illustrates our proposed mechanism of how two separate images of a visual scene from each retina are imperceptibly and instantaneously combined into a single seamless image within the mind. The simplified visual pathway illustrates how light from the scene projects onto each retina, including the aligned foveae, and is processed via the visual pathways. The separate monocular images converge within the primary visual cortex, are processed within corticothalamic feedback loops and the thalamus integrates the information from these feedback loops. Although the retinas are intrinsically wired for distance, the parietal lobes create a spatial map of the visual scene as well as a spatial map of the body and other sensory information. When this processed visual and other sensory information, including the spatial mapping, is integrated by the thalamus it makes the 3D default space. We propose that the final processed image that arises to conscious awareness is filled-in by the thalamus and is experienced at the eyes. (Figure and figure description reproduced with permission from copyright holder. Figure by Lynsey Ekema, MSMI).

**Figure 4:**
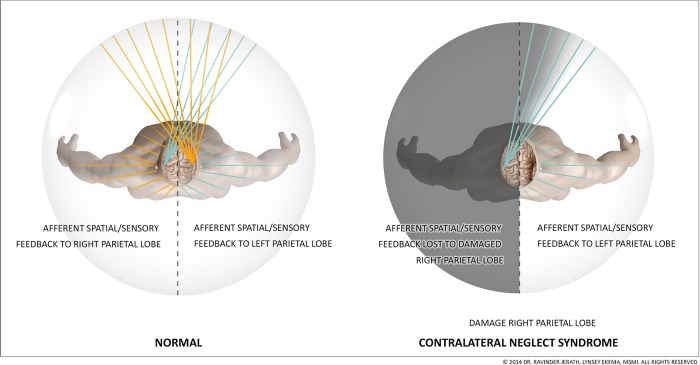
**Connections of the left and right parietal cortex: visual fields and body space**. The figure depicts how the right parietal lobe, in a healthy individual, receives visual information from the left eye, and sensory information from the left side of the body while the left parietal lobe receives sensory information from the right eye and right side of the body. The parietal lobes spatially map this information so that the mind can spatially locate stimuli. In contralateral neglect syndrome, when the right parietal lobe is damaged, information is fed to both parietal lobes, like in a healthy individual, but the intact left parietal lobe is able to spatially locate visual and sensory stimuli while the damaged right parietal lobe is not. The external visual information and internal sensory information sent to the damaged right parietal lobe does not become spatially oriented, like it does in a healthy individual, so this information never becomes a part of conscious awareness within the 3D default space. This results in a patient not being able to see or feel stimuli on their left side, even though their eyes, occipital lobe, and nerves are functioning normally. (Figure and figure description reproduced with permission from copyright holder. Figure by Lynsey Ekema, MSMI).

Our consciousness model can help to explain various conditions such as contralateral neglect syndrome, phantom limb syndrome, motion sickness, and vertigo, as well as phenomenon such as blindsight and scotomas. The 3D default space is a large space that is filled-in with processed sensory information that has been intricately mapped by parietal networks (Figure 3). This internal space recreates the outside world within the mind. In a previous paper, we discussed insights about consciousness by examining contralateral neglect (CN) syndrome. CN is most commonly caused by lesions within the right parietal lobe. Patients with CN have intact eyes and nervous systems but they are unable to see or locate objects within external space on their left sides (Kerkhoff, 2001) and some patients are not even able to feel sensations on the left side of their body (Kerkhoff, 2001) (Figure 4). This disorder is well-documented clinically and numerous studies on the disorder provide insights into consciousness and how external space is recreated internally within the mind (Jerath and Crawford, 2014). We propose that both visual and non-visual information from the left side is lost in the damaged right parietal lobe and cannot be integrated by the thalamus within the 3D default space (Jerath and Crawford, 2014).

**Figure 5:**
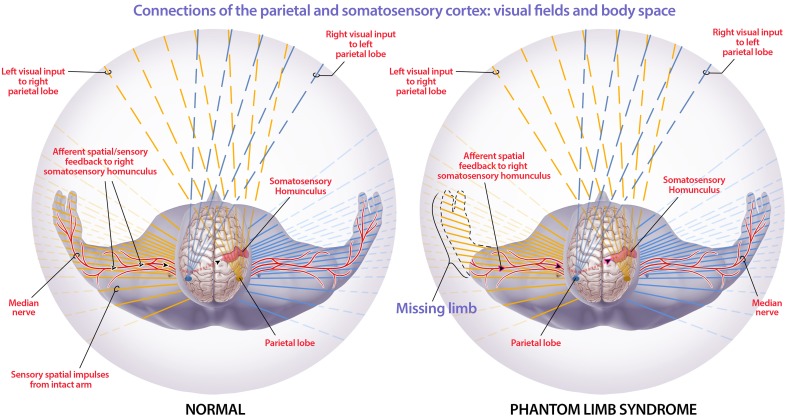
**Connections of the parietal and somatosensory cortex: visual fields and body space**. The orange lines are visual and non-visual information sent to the right parietal cortex while the blue lines are visual and non-visual information sent to the left parietal cortex. The red sensorimotor cortices are depicted in each hemisphere and the corresponding red lines illustrate the processed sensorimotor information that is filled in within 3D default space. The figure depicts how the right parietal lobe and sensory motor cortex, in a healthy individual, receives visual information from the left eye, and sensory information from the left side of the body. The parietal lobe spatially maps this information so that the mind can spatially locate stimuli. In phantom limb syndrome, when the left arm has been amputated, visual information that the limb is gone is received by the left eye and processed so that the visual information filled into the 3D default space does not include a left arm within the visual space. Though there is no longer sensorimotor information flowing from the now amputated limb to the cortex, the sensorimotor and other networks in the brain are still intact. This leads to intrinsically-generated sensations that are integrated within the 3D default space, generating an intact non-visual representation of the limb and phantom limb sensations. Phantom limb pain is likely due to the integration of this contradictory sensory information and subsequent representation within 3D default space. The solid lines extending through the body illustrate the information, from the corresponding cortex, that is filled into the 3D default space. (Figure and figure description reproduced with permission from copyright holder. Figure by Michael Jensen MSMI, CMI).

Our model may also provide insights into phantom limb syndrome and phantom limb pain. In our consciousness model, consciousness and body schema arise when sensory information from throughout the body is processed by corticothalamic feedback loops and integrated by the thalamus (Jerath and Crawford, 2014). We propose that phantom limb syndrome arises when a limb is lost but the neural circuits within the brain, such as somatosensory and motor homunculi, remain intact (Figure 5). This allows for phantom sensations to arise from within the brain without afferent signaling from the limb. In addition, phantom pain is likely the result of the integration of conflicting sensory information within the 3D default space, such as the visual information that the limb is gone accompanied by the phantom sensations that the limb is still intact. Furthermore, cortical reorganization, in which nearby somatosensory areas invade the amputated limb somatosensory area (Ramachandran and Hirstein, 1998; Flor et al., 2006), can result in further conflicting sensory information where phantom limb sensations are felt on other body areas such as the face (Ramachandran and Hirstein, 1998).

Motion sickness and vertigo are two more conditions that may be explained by our model. Both motion sickness and vertigo are likely the result of the integration of conflicting sensory information by the thalamus within the 3D default space. In the case of motion sickness, the visually perceived movement and the vestibular systems perceived movement conflict with each other. Similarly, the most common cause of vertigo is from free-floating calcium crystals called “otoconia” that make their way into the semicircular canals and distort the sense of movement so that actual head movements, vision, and sensory information from the vestibular system contradict each other and cause a spinning sensation or dizziness. Again, the integration of conflicting sensory information results in the sensation of dizziness.

Similar supporting evidence is provided by studies on blindsight. Patients with this condition are cortically blind due to lesions in the striate cortex but they demonstrate unconscious visual awareness, especially when doing forced-choice procedures. These blind patients show a much higher accuracy of identifying objects than from chance alone. Some researchers propose that this phenomenon is due to possible pockets of healthy tissue within the damaged striate cortex, which may lead to enough visual processing for blindsight but not conscious awareness (Fendrich et al., 1992). Others propose that blindsight can occur, in the presence of damage to the striate cortex, because of the lateral geniculate body (LGN) in the thalamus (Schmid et al., 2010). The underlying mechanism of blindsight may be similar to that of CN syndrome that results from parietal lobe damage. In blindsight patients, we propose that visual information is processed within the brain but the information is not able to be processed within the damaged striate cortex and it is therefore not able to be integrated by the thalamus into the 3D default space. The visual information is unconsciously processed but does not rise to conscious awareness, similar to in CN.

Studies on scotomas or blind spots may also support our 3D default space consciousness model. During visual processing, scotomas are filled in within a neural representation and a seamless visual image, lacking blind spots, is perceived (Ramachandran and Gregory, 1991), supporting the proposition of the creation of an internal neural representation such as the visual space of the 3D default space. The phenomenon of afterimages also supports a consciousness mechanism involving the creation of a neural visual representation and filling-in process. Both negative and positive afterimages involve higher level cortical and filling-in processes (Shimojo et al., 2001; Holcombe et al., 2004; Sperandio et al., 2012), challenging the previous idea that these afterimages were the result of over-stimulated photoreceptors in the retina (Brindley, 1962). However, some researchers still propose that these afterimages are generated within the retina (Zaidi et al., 2012).

## Homeostasis and slow oscillations

We propose that the sensory memory space of larger 3D default space is likely generated by oscillations emanating from the spinal cord, brainstem, reticular activating system, default mode network (DMN), and other salient networks (Jerath and Crawford, 2015b). This active but idling space is then filled-in with processed sensory information from throughout the entire body, recreating both visual and non-visual space. The body plays a significant yet rarely recognized role in consciousness processes. Constant afferent sensory feedback from the body, which helps maintain homeostasis, is processed within the brain and forms the non-visual portion of the filled-in 3D default space. Some of this non-visual sensory information remains unconscious, but may still lead to homeostatic changes throughout the body, while other non-visual sensory information rises to conscious awareness. This results in conscious experiences of sensations such as smelling a scent, hearing a sound, or feeling hunger pains, or thirst.

The body exhibits slow and infra-slow physiological oscillations ranging from 1 Hz to < 0.01 Hz (Hiltunen et al., 2014). Cardiorespiratory oscillations integrate with brainstem pacemakers and salient networks, including the DMN, modulate neural, and autonomic nervous system activity, and may lead to widespread cellular membrane potential changes (Jerath et al., 2014a). We also propose that these membrane potential changes may occur with each inspiration and expiration (Jerath et al., 2014b). The glial syncytium maintains a high resting state membrane potential, ranging from −80 to −90 mV (Verkhratsky and Butt, 2013). Astrocytes generally have membrane potentials around 20 mv higher than surrounding neurons (Somjen, 1995). We propose that the higher resting membrane potential of astrocytes may be involved in the maintenance of the membrane potentials of surrounding neurons. For example, astrocytes likely regulate concentrations of extracellular potassium via spatial buffering, as well as carrier-operated and channel-operated potassium chloride uptake (Walz, 2000). When a neuron discharges this leads to increases in extracellular potassium concentrations, which can then interfere with neuronal signaling by causing further neuronal depolarizations. Astrocytes take up this excess potassium, which leads to a local depolarization which results in the dispersal of potassium into the bloodstream. This allows for normal neuronal action potential propagation to continue. In addition, widespread membrane potentials of neural and non-neural cells throughout the body may be maintained by the cardiorespiratory system (Jerath et al., 2014a).

These slow oscillations dynamically synchronize and interact and may be involved with energy metabolism. The physiological and homeostatic interactions of all body tissues and cells, provide energy to neurons, astrocytes, and form an active infrastructure in which transmissions from peripheral areas of the body reach the thalamus and are processed within corticothalamic feedback loop within milliseconds. For example, neural networks within the corticothalamic system process and perceive visual sensory information within 150 milliseconds (Thorpe et al., 1996). It is well-known that during disorders of homeostasis, such as fluid imbalance (dehydration), brain metabolic activity is inefficient and prolonged dehydration may negatively affect executive functioning (Kempton et al., 2011). Other homeostatic disruptions include deviations from optimum oxygen levels and calcium homeostasis. For example, hypoxia induces neuronal cell death in the brain (Malhotra et al., 2001) and leads to many different negative effects throughout the body (Pierson, 2000). Even too much oxygen can lead to negative effects such as oxygen toxicity (Stogner and Payne, 1992). Disruption of normal calcium homeostasis can lead to hypercalcemia or hypocalcemia, both of which have negative effects on the body. Any imbalances in ionic homeostasis can lead to major physiological ailments including loss of consciousness and are known to underlie neurological disorders such as epilepsy (Rogawski, 2000).

## Layers of neural activity

We propose that neural activity consists of distinct but highly interactive layers of oscillatory activity. The first layer of neural activity consists of the oscillatory activity from the default mode network, other resting state networks, cardiorespiratory system, and ascending reticular activating system (Jerath and Crawford, 2015b). These oscillations likely support all other neural activity by creating an idling state in the brain on which other oscillatory activity can build, as well as creating a unified space via slow oscillatory activity. The majority of the energy utilized by the brain can be attributed to the DMN (Fox et al., 2005) and 90% of the energy used by the brain can be attributed to intrinsic activity rather than activity provoked by stimuli (Raichle and Snyder, 2007), making the DMN a prime candidate for the baseline neural activity. These oscillations also likely form the active sensory memory space in which the thalamus fills in processed sensory information. The sheer amount of energy utilized by intrinsic neural activity and the involvement of the default mode network and brainstem in consciousness suggest that default mode activity and other intrinsic activity may underlie higher processing and activity in the brain (Jerath and Crawford, 2015b). It has been previously proposed that intrinsic neural activity may maintain the framework of a spatiotemporally oriented virtual neural space which helps to organize incoming sensory information (Northoff, 2013). However, it is extremely difficult to determine cause and effect relationships underlying this oscillatory activity or identify a neural “space” from neural activity; therefore, further research is needed to elucidate this possible mechanism. We propose that this slow-wave and synchronization activity of the first neuronal layer is an essential part of consciousness. For example, sensory stimulation activates the ascending reticular activating system projections to the thalamus which then activate the cortex and trigger descending signaling to the spinal cord (Garcia-Rill et al., 2013). This same system is also involved in the lack of sensory awareness during slow-wave sleep and the atonia of REM sleep, which prevents us from acting out dreams (Garcia-Rill et al., 2013). Synchronized episodes of neural activity throughout the brain may constitute the electrical signatures of thoughts and behaviors (Thiagarajan et al., 2010). Modafinil, a drug used to promote wakefulness, increases electrical coupling in the reticular activating system via gap junctions. It reduces reticular activating system activity and the release of gamma amino-butyric acid (GABA), which disinhibits other neural systems and increases coherence within the reticular activating system (Garcia-Rill et al., 2007) and corticothalamic system (Urbano et al., 2007). These findings demonstrate the important role of synchronization and the ascending reticular activating system and thalamus in consciousness. Other specifics regarding how this first layer of neural activity functions requires further research.

Normal thalamocortical oscillatory activity can range from infra-slow to ultra-fast and is generated by short and long range synchronizations (Timofeev et al., 2012). Neural synchronization may be generated by varying degrees via chemical synaptic mechanisms, gap junction activity, ionic fluctuations, and ephaptic coupling (Timofeev et al., 2012). Some studies have suggested that infra-slow oscillations may synchronize faster neural oscillations (Vanhatalo et al., 2004) and infra-slow oscillations have been shown to be highly correlated with faster oscillations ranging from 1 to 40 Hz, suggesting a relationship between fast and slow oscillations (Monto et al., 2008). Another study found a significant correlation between alpha EEG power, respiration, and BOLD signals during resting, suggesting a relationship between this oscillatory activity as well as a possible common neuronal origin (Yuan et al., 2013). In addition, some researchers have proposed that the neuronal cytoskeleton, which has been shown to support energy transport and information processing, may be involved in establishing dynamic synchronized brain states that may modulate connectivity (Plankar et al., 2013). Synchronized oscillatory activity has been shown to be involved in many cognitive processes and abnormal neural synchronization may underlie many brain disorders (Uhlhaas and Singer, 2006). We propose that the first layer of neural activity is involved this synchronization process, creating a framework on which faster oscillatory activity builds, and creating a dynamic neural sensory memory space (Jerath and Crawford, 2015b).

**Figure 6:**
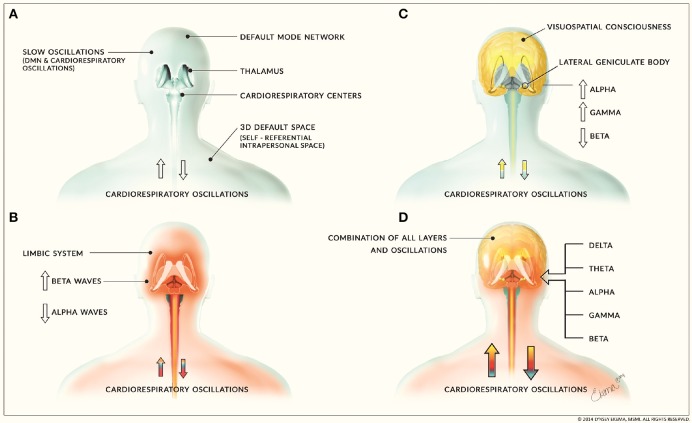
**Layers of neural activity**. **(A)** depicts the first layer of neural activity that includes slow oscillations from the default mode network and cardiorespiratory oscillations. This forms the basis for all other layers of neural activity and is depicted by the blue coloring. **(B)** includes limbic activity and is depicted by the red coloring. It is associated with increased beta waves and decreased alpha waves. **(C)** includes corticothalamic feedback loops involved in cognitive and consciousness processes and is depicted by yellow coloring. This layer is associated with increased alpha and gamma waves and decreased beta waves. These layers combine to form **(D)**, the sum of human neural activity consisting of all neural and physiological oscillations. The combination of this activity is denoted by the combination of the blue, red, and yellow layers. (Figure and figure description reproduced with permission from copyright holder. Figure by Lynsey Ekema, MSMI).

The second layer of neural activity consists of limbic oscillations and cardiorespiratory oscillatory activity while the third layer consists of higher cognitive processes involving corticothalamic feedback loops and consciousness processes. It has been proposed that the limbic system may have evolved before higher cortical areas (Holden, 1979) which is consistent with our classification of these neural activity layers. This layer of higher cortical activity and oscillations are responsible for the corticothalamic processing that fills in the 3D default space. Emotions and feelings, arising from the limbic system layer, also fill in this space. These three layers form the sum of human neural oscillatory activity (Figure 6). This proposed structuring of neural activity helps to further clarify how our consciousness mechanism works.

We propose that certain oscillations are associated with specific layers of neural activity; however, these oscillations are not confined to any one layer. We propose that the first layer of activity is composed of slow oscillations and delta waves. The default mode network of the first layer consists of spontaneous low frequency oscillations < 0.1 Hz and respiratory, cardiac, and blood pressure oscillations. These oscillations have been shown to synchronize at 0.15 Hz at rest which may be due to decreased activity of reticular neurons in the brainstem. In addition, cholinergic neurons in the pedunculopontine nucleus (PPN) of the brainstem fire in phase with slow oscillations during slow-wave sleep and modulate cortical gamma activity (Mena-Segovia et al., 2008). Delta oscillations are the predominant frequency of waking reptiles and are prominent during early development in humans and slow-wave sleep, suggesting that delta oscillations may be associated with more ancient neural systems (Knyazev, 2012). Delta oscillations may also be involved in integrating homeostatic processes and cerebral activity (Knyazev, 2012). In addition, functional organization of respiratory, cardiac, and delta-theta related rhythms is dependent on coupling between cortical and reticular formation delta and other low frequency oscillations (Lambertz and Langhorst, 1998). However, reticular activating neurons of the PPN and intralaminar parafascicular nucleus of the thalamus also exhibit electrical coupling and fire at beta/gamma frequencies when maximally activated (Garcia-Rill et al., 2013), therefore activity in other frequency bands is also observed in this and other layer.

The second layer or limbic layer is comprised predominantly of beta and theta oscillations. For example, active brain states are associated with hippocampal theta oscillations (Saper et al., 2010) and electrical stimulation of limbic areas evokes theta oscillatory activity (Gray, 1982). Neural activity in lower mammals is dominated primarily by theta oscillations (Knyazev, 2012), which may support the proposed role of theta activity in the secondary layer. Negative emotions are associated with increased beta responses (Güntekin and Başar, 2010) and beta oscillatory activity is associated with motor control and is observed in the basal ganglia (Bogacz, 2014). However, beta oscillations are also observed in cortical sensorimotor (Kilavik et al., 2013) and visual areas (Engel and Fries, 2010); therefore, the exact role of beta oscillations within these layers requires further study. It has been proposed that beta oscillatory activity is involved in maintaining current sensorimotor and cognitive states so that beta activity is enhanced if maintenance of the current state is intended or predicted (Engel and Fries, 2010) and pathological increases in beta activity, such as that seen in Parkinson's disease, decreases cognitive control (Engel and Fries, 2010). Lastly, the third or corticothalamic layer consists primarily of alpha oscillatory activity which has been associated with cognitive processing and access to memory, and may have an inhibitory function (Klimesch, 2012). Along with gamma oscillatory activity which is associated with cognitive processing, perception, and memory (Bosman et al., 2014).

## Memory

Consciousness processes and memory processes are closely linked and highly interactive. In fact, the reproduction of external and internal space within the mind during consciousness processing is supported by the phenomenon of memory. We propose that memories cannot be made unless external and internal space is reproducible within the mind. This reproduced information must then be encoded, stored, and then it can be retrieved back into the active sensory memory space of the 3D default space. For example, researchers have proposed that each time a memory is revisited the memory is re-encoded enhancing conceptual aspects which diminish contextual details and sometimes add false information (Yassa and Reagh, 2013). We propose this is because the memory is retrieved and represented within the active sensory memory space of the 3D default space where it is then re-encoded to be stored again.

Sensory impulses can be both externally activated and internally generated. For example, sensory information from end organs would constitute externally activated sensory content while inner speech and mental imagery are internally activated. A recent study has shown that mental imagery processing likely involves top-down processing, a reversal of the direction of information flow from visual perception (Dentico et al., 2014). This reversal of the predominant direction of cortical signal flow has also been proposed to underlie the phenomenon of dreaming (Nir and Tononi, 2010). In addition, this reversal may disrupt the usual formation of new memories and may be responsible for dream amnesia (Nir and Tononi, 2010). The phenomenon of dreaming helps to illustrate our concept of the thalamus creating an internal representation of external space within the mind. In our model, mental imagery, inner speech, dreaming, and other internally activated sensory content likely occurs via top-down processes that still involve integration by the thalamus in order to rise to conscious awareness. We propose that the sensory memory space is a neural space within 3D default space in which conscious experience, which is represented by the thalamus, can become encoded and stored as memories that can be retrieved and brought back into this sensory space during recollection. This is why we can visualize a place or an event and remember the extensive details and layouts of that scene. We have discussed the important role of the thalamus in consciousness and many studies have demonstrated the important role of the thalamus in memory processing. For example, the anterior thalamus has extensive connections to the hippocampus, involving the mammillary bodies, retrosplenial cortex, and parts of the prefrontal cortex that form memory pathways (Aggleton et al., 2010). In addition, the thalamus, including the mediodorsal nucleus and anterior nuclei are critical for recognition memory by recall during encoding and retrieval (Pergola et al., 2013). The anterior cingulated cortex, thalamus, and cerebellum all show high activation during recollection (Cabeza et al., 1997). Another study showed that patients with thalamic damage had deficits in long-term memory while patients with damage to the mammillothalamic pathway experienced deficits in short-term memory (Van Der Werf et al., 2003).

A study on visual memory in CN patients had patients in Milan describe the Piazza del Duomo, a well-known public square. The patients were asked to imagine and describe the square from two different viewpoints. From the first viewpoint, the patients neglected to describe places on the left side of their field of view, which is consistent with the neglect seen in CN. However, when describing the square from the second viewpoint, the patients no longer included landmarks and places on the left side that they had just described in the first view (Bisiach and Luzzatti, 1978). This study demonstrates the possible role of the 3D default space as both an active sensory memory space and a space where memories are recalled. In CN, patients do not consciously see stimuli in their left visual field and in some cases cannot feel stimuli on the left side of their body. These CN patients had seen this square before developing CN, but their recollection of the memory of the place was affected by the parietal lesions. When the patients visualized the layout of the square and then shifted their perspective to a new vantage point they could no longer visualize those places that they had just described a moment before because they were now in their left visual field, We propose that this is because the intact memories are recalled within this active sensory memory space for recollection but the damaged parietal lobe cannot spatially locate the stimuli on the left side and it is lost from conscious awareness.

## Coma

The main indicator of a comatose state is the absence of arousal and in turn the absence of consciousness. Comas are caused by a disturbance in the reticular activating system and thalamic projections. Thalamic lesions can lead to coma and paroxysmal sleep (Bjornstad et al., 2003), demonstrating the important role of the thalamus in consciousness. People in comas do not experience normal sleep wake cycles. Comatose patients lack both awareness and wakefulness while patients in a vegetative state are in a state of partial arousal and lack true awareness. A recent study that compared the neural activity of 32 patients with chronic disorders of consciousness with 26 healthy controls found that alpha networks in some of the patients, considered to be in unresponsive vegetative states, actually exhibited well-preserved alpha networks (Chennu et al., 2014). One patient in particular demonstrated alpha band networks very similar to healthy controls, including long-range connections between occipital, parietal, and frontal regions of the brain (Chennu et al., 2014). Studies like this can help us to recognize brain activity that is indicative of conscious awareness in vegetative patients that do not demonstrate physical responses but may exhibit responsive neural activity when asked to imagine a scene. In addition, studies involving vegetative patients have demonstrated improvements in patient responsiveness following thalamic stimulation; however, definitive evidence could not be drawn from these studies because it cannot be determined with certainty whether these improvements were related to the thalamic stimulation or spontaneous recovery (Shah and Schiff, 2010). However, a case study on a patient who had been in a coma for 6 years due to a traumatic brain injury, found that thalamic stimulation resulted in the patient exhibiting responsive behaviors to sensory stimulation directly related to the stimulation (Schiff et al., 2007). There are various proposals regarding the possible mechanisms underlying the effects of thalamic stimulation involving activating arousal systems and introducing excitatory activity that may restore some normal awake state neural activity (Shah and Schiff, 2010). The use of thalamic stimulation as a possible treatment in coma patients demonstrates the important role of the thalamus in arousal and consciousness processes.

## Slow-wave sleep

The thalamus, with its reciprocal fast connections to the cortex during the awake state, forms the basis of cognition in a conscious individual. During slow-wave sleep the thalamus shifts from tonic firing to burst mode. Tonic firing during awake states transmits information from the thalamus to the cortex while burst mode firing is likely involved in the disconnection of the thalamus and cortex and subsequent unconsciousness (Purves et al., 2001). Conscious experience is based on widespread membrane potential changes that occur during both waking and sleep states. In fact, widespread membrane potential changes may underlie the restorative properties of sleep (Jerath et al., 2014c). Tononi has proposed a similar hypothesis in which the function of sleep is to reestablish synaptic homeostasis after an increase in synaptic strength that occurs during wakefulness (Tononi and Cirelli, 2003). However, we go a step further and propose that the slow-wave activity involved in this downscaling is due to progressively increased levels of cardiorespiratory coherence during stages of slow-wave sleep that lead to changes in sympathovagal balance (Jerath et al., 2014c). This mechanism is consistent with our current comprehensive model in which high levels of cardiorespiratory coherence may modulate the autonomic nervous system and cause a shift toward parasympathetic dominance during meditative states (Jerath et al., 2014a), as well as the possible direct effects of respiration on widespread membrane potential changes (Jerath et al., 2014b). In addition, during slow wave sleep, disconnection between the thalamus and cortex occurs and activity of the thalamus and cortex decrease (Magnin et al., 2010). There is also reduced corticothalamic connectivity during general anesthesia (White and Alkire, 2003) and thalamic metabolism and blood flow are also reduced (Alkire and Miller, 2005). These studies emphasize the important role of the thalamus and corticothalamic connectivity in consciousness. In addition, thalamic deactivation usually precedes cortical deactivation at the onset of slow-wave sleep. This likely underlies the experience of hallucinations that can occur during this transition, as well as the common over-estimation of the time taken to fall asleep (Magnin et al., 2010). In our model, the oscillations that create the 3D default space and corticothalamic feedback loops orient us to time and space, therefore this deactivation and disconnection of the thalamus followed by the cortex can result in hallucinations (distortions of visual space) and distortions in the perception of time. This study demonstrates the importance of corticothalamic interactions in consciousness as well as how corticothalamic interactions can affect the perception of time within the 3D default space.

## REM sleep and dreaming

Examining dreaming, as well as contrasting dream states with waking states can contribute novel insights to the study of consciousness (Windt and Noreika, 2011). Dreaming consists of a unique state of consciousness in which the brain is in a closed system and does not experience external input from the environment. Dreams consist of subjective experiences that depend solely on already stored information in the brain (D'Agostino et al., 2013). Many researchers propose that dreaming does not involve reflective-awareness. Although, it is likely that the same levels of reflective-awareness that are present during waking states are not present during dreaming, it would be inaccurate to claim dream states lack self-awareness altogether. For example, emotions and feelings of self-consciousness are common experiences in dreams (Kahan and Laberge, 1994).

Lucid dreaming is dreaming in which one is aware that one is dreaming and is able to exert some control over the content of the dream (Holzinger et al., 2006). In non-lucid dreaming, a person is unable to distinguish whether or not the experience is actually occurring. In addition, people with mental disorders, involving hallucinations, experience abnormal awareness during waking consciousness because they are unable to distinguish between externally and internally generated percepts, similar to what occurs in non-lucid dream states (D'Agostino et al., 2013). It is interesting to note that many mental and mood disorders also experience disorder-specific dreams (Beauchemin and Hays, 1995; Cartwright et al., 2006; Lusignan et al., 2009). By examining dreams, we can gain insights into how neural activity translates into conscious experience (D'Agostino et al., 2013). When compared to non-lucid dreaming, lucid dreaming is associated with higher levels of beta-1 frequency band oscillations in both parietal regions (Holzinger et al., 2006). Lucid dreaming consists of features indicative of both waking and non-lucid dreaming states of consciousness (Voss et al., 2009). Waking is characterized by high alpha band coherence while lucid dreaming is characterized by increased theta and delta coherence (Voss et al., 2009). This theta and delta coherence is highest in the frontal cortex and likely involves higher activation in the dorso-lateral prefrontal cortex (DLPFC) (Voss et al., 2009). REM sleep is usually associated with decreased activation in the DLPFC, which may explain the reduced self-awareness that occurs during non-lucid dreaming (Muzur et al., 2002), Voss *et al*. proposes that what Edelman has termed “secondary” consciousness is activated during lucid dreaming, which accounts for the similarities of neural activity experienced during lucid dreaming and waking states (Voss et al., 2009). According to Edelman, primary consciousness consists of a present multimodal scene while secondary consciousness involves various other features including reflection, abstract thought, and consciousness of being conscious (Edelman, 2004). Edelman proposes that primary consciousness occurs during non-lucid dream states while secondary consciousness occurs during lucid dream states (Edelman, 2004); however, Foulkes proposes that the ever-present characteristic of dreaming, in which an imagined scene is integrated and constructed by long-term memory, likely involves secondary consciousness processes (Foulkes, 1999). The generation of internally-generated scenes is present in both non-lucid and lucid dreaming, suggesting the involvement of secondary consciousness characteristics in both non-lucid and lucid of dreaming.

## Role of respiration and emotions

**Figure 7:**
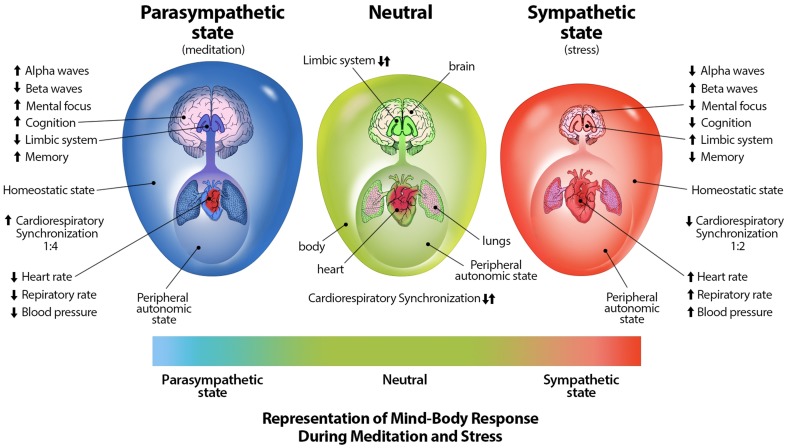
**Representations of mind-body response during stress and meditation**. The figure illustrates the spectrum of emotion states and corresponding physiological states. At the far end of the spectrum is a stressed sympathetic state. The largest encompassing circle represents the entire body and homeostatic state, while the large circle inside represents the afferent signaling from the body. At the left end of the spectrum, in the parasympathetic meditative state, the heart is smaller to depict decreased heart rate, and increased cardiorespiratory coherence. The heart and brain are depicted connected to illustrate the increased synchronization and coherence that occurs in more calm states. The brain is large to show increased cognitive abilities during this state and the orb representing the afferent signals from the body is smaller in size to depict less chaotic and more synchronized signaling. At the far right end of the spectrum, in this stressed sympathetic state, the afferent signaling from the body is larger to depict how the brain is dominated, and distracted by powerful and chaotic afferent signaling throughout the body. The heart is also larger to show the increased heart rate during this state and decreased cardiorespiratory coherence. The brain is smaller to illustrate the decreased cognitive abilities during anxiety and stressed states. In addition, during this state, the brain and heart are depicted as separate because many neural, and physiological oscillations are desynchronized during this state. Overall, a relaxed mind is associated with decreased excitation and cardiorespiratory coherence while a stressed or anxious mental state, with highly activated emotion centers in the brain, is associated with more desynchronized cardiorespiratory rhythms and high excitation throughout the body. (Figure and figure description reproduced with permission from copyright holder. Figure by Michael Jensen MSMI, CMI).

Both neural impulses and physiological feedback play a significant role in the experience of emotions. In fact, respiration and emotions are so correlated that respiration (Bloch et al., 1991) and cardiorespiratory activity (Rainville et al., 2006) can be used to distinguish between different emotions. The medulla and pons, where respiratory rhythms are generated in the brain (Feldman and Del Negro, 2006), have efferent and afferent connections with the limbic system (Homma and Masaoka, 2008; Evans, 2010). A study examining increased respiration rates and anxiety detected respiration-related anxiety potentials in limbic areas of the brain following inspiration (Masaoka and Homma, 2000). These studies further illustrate the strong relationship between emotions and respiration. The modulation of emotions by respiration can be utilized as a possible treatment of mood disorders, and to help reduce stress and enhance feelings of well-being. By practicing slow, deep breathing such as pranayama or meditation people may be able to actively influence their mental and emotional state. We have proposed that cardiorespiratory and default mode network oscillatory activity, which make up idling activity of the brain, likely underlie faster oscillations involved in higher processing (Jerath and Crawford, 2015b). The relationship between respiration and emotions further emphasizes the important role of respiration in influencing both unconscious and conscious neural activity and further highlights the influence of the body on the mind. Too often, consciousness models neglect the significant signaling and feedback from the body that influences neural activity; our model recognizes these inputs and incorporates them in our proposed mechanism. We propose that the autonomic nervous system is likely modulated by increased levels of cardiorespiratory coherence that occur during slow, deep breathing, and meditation (Jerath et al., 2012) (Figure 7).

## Mindfulness meditation and pranayama

The central role of widespread membrane potential based homeostasis can also explain how slow deep breathing, such as in *pranayama*, can lead to autonomic modulation. This topic and the possible underlying mechanisms have been discussed in detail in our previous articles (Jerath et al., 2006, 2012). Changes in mental state, from anxiety to a meditative or relaxed state, are due to a total body response with associated changes in cardiorespiratory, peripheral, and DMN oscillations (Jerath and Barnes, 2009). This mechanism is at play during awake states, when memory, attention, and cognitive skills can be enhanced by meditation (Mrazek et al., 2013). This underlying meditation mechanism is similar to but less pronounced than the previously discussed mechanism that underlies the widespread hyperpolarization that occurs during slow wave sleep (Jerath et al., 2014c). This may also be associated with the membrane potential driven disconnection of thalamus from the cortex may be involved in sleep. One study found that disconnection of thalamic neurons from the cortex resulted in a hyperpolarization of around 9 mv, as well as the generation of spontaneous slow oscillations (Dossi et al., 1992).

**Figure 8:**
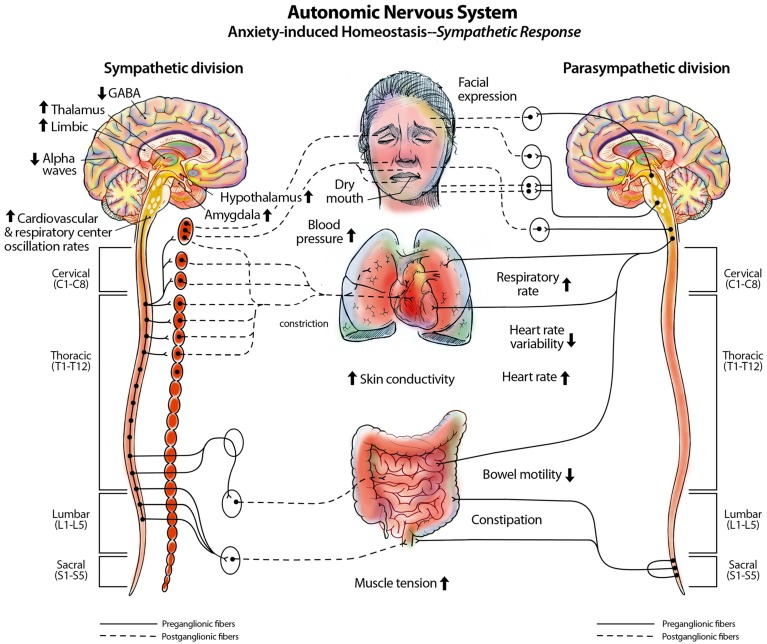
**Responses associated with sympathetic dominance**. The dual-sided autonomic nervous system is shown in which sympathetic activation outweighs the parasympathetic branch. Sympathetic dominance, as seen during the stressed state, leads to a variety of peripheral responses associated with depolarization of target organs. There is an increase in DMN activity and mind wandering. Functional connectivity and alpha waves decrease, while limbic and amygdala activity increase. Brainstem activity also increases, reflected by the increased respiratory center and cardiovascular center rates. Respiration and heart rate increase. Heart rate variability measures are dominated by low frequency components. Blood pressure, skin conductivity, and muscle tension increase. Bowel motility and salivary gland secretion are also hindered. The relatively depolarized state is illustrated by red shading throughout the various target organs and spinal cord. Facial expression depicts a stressed or unpleasant state. (Figure and figure description reproduced with permission from copyright holder. Figure by Michael Jensen MSMI, CMI).

**Figure 9:**
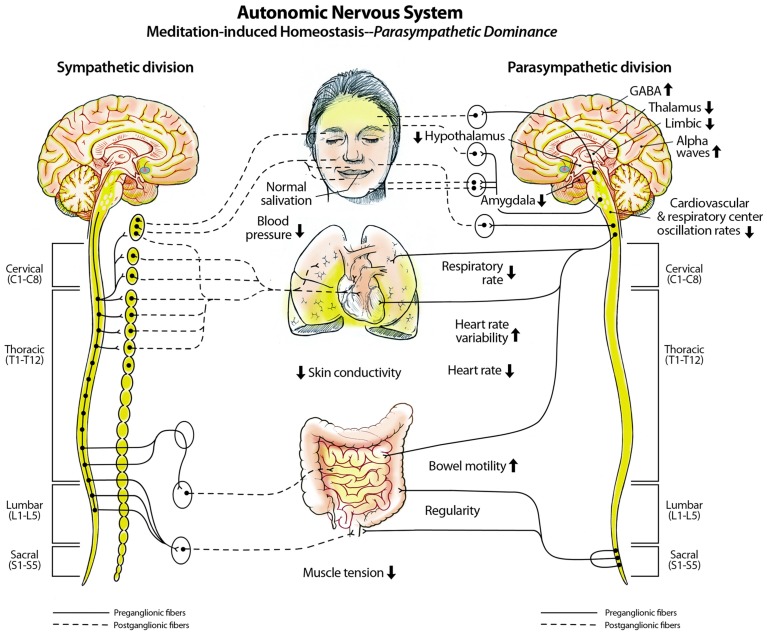
**Responses associated with parasympathetic dominance**. The dual-sided autonomic nervous system is shown, in which parasympathetic activation outweighs the sympathetic branch. In contrast to sympathetic dominance, a shift to prominent parasympathetic activation leads to peripheral responses associated with membrane potential hyperpolarization and the reversal of the effects seen in Figure 8. In this state, DMN activity and mind wandering decrease, leading to more mental focus and increased memory. There is an increase in functional connectivity and alpha waves and a decrease in limbic and amygdala activity. Brain stem activity decreases, which is reflected in decreased respiratory center and cardiovascular center rates. Heart rate, respiratory rate, blood pressure, and muscle tension all decrease. High frequency heart rate variability is increased. This parasympathetic shift can be readily achieved through meditation and deep breathing techniques. A pleasant facial expression is associated with this state and yellow shading throughout the figure help illustrate hyperpolarization, compared to the red color of the relatively depolarized state in Figure 8. (Figure and figure description reproduced with permission from copyright holder. Figure by Michael Jensen MSMI, CMI).

Many known observations during meditation such as an attainment of calmness, feeling of oneness with the universe may be due to sub-conscious respiration-induced low and ultra slow frequency rhythmic changes in membrane potential throughout the body. In a previous paper, we correlated respiration with widespread membrane potential changes, specifically widespread depolarization during expiration and repolarization during inspiration (Jerath et al., 2014b). In this model, we go a step further to propose that these widespread membrane potential changes that may occur as a direct result of inspiration and expiration are enhanced by slow deep breathing and increased cardiorespiratory coherence and these membrane potential changes result in the slow oscillatory activity involved in creating the framework of the 3D default space. The correlation of respiration to widespread depolarization and repolarization has been previously proposed (Jerath et al., 2014b). In addition, the central role of global membrane potential based homeostasis can also explain how slow deep breathing, such as that practiced during *pranayama* and meditation, can lead to autonomic modulation. For a detailed look at this phenomenon (see Jerath et al., 2006, 2014c; Jerath and Barnes, 2009). The change in mental state from a sympathetic stressed state to a parasympathetic mind-body response is based on total body response with associated changes in cardiorespiratory, peripheral, and DMN oscillations (Jerath and Barnes, 2009) (Figures 8, 9). This is likely a less pronounced but similar mechanism to what occurs during slow-wave sleep, in which widespread hyperpolarization occurs throughout the cortex and subcortical structures (Steriade and Timofeev, 2003; Jerath et al., 2014c).

## Conclusion

The most currently accepted consciousness theories offer insights into consciousness processing but they fail to address many key aspects of conscious experience. We propose that our unified consciousness model addresses these neglected aspects of consciousness (Table 1). Our model involves the creation of a neural sensory memory space from oscillatory activity from the default mode network, other salient networks, spinal cord, brainstem, and reticular activating system. This idling brain activity may provide the foundation for an active space that is filled-in by the thalamus. We propose that external and internal sensory information is processed by corticothalamic feedback loops and projected by the thalamus within a proposed 3D default space, recreating the external world within the mind. This 3D default space is composed of all the interconnected cells of the body, which act as one unified space (Table 2). Within this larger intrapersonal space is the neural sensory memory space in which moment-to-moment subjective experience occurs, including hallmarks of conscious experience such as visual imagery, thoughts, and inner speech. Current technology and current understanding of the brain limit our ability to verify these consciousness models but rapid advances in the field show promise. Processing by corticothalamic feedback loops, extremely fast processing times, and the subjective nature of first-person conscious experience make consciousness a difficult topic to study and pinpoint where, when, and how consciousness arises. However, comparing neurophysiological and neuroimaging from minimally contrasted experimental data of unconscious and conscious processing can inform better choices for study of objective measures of the emergence of conscious (Dehaene and Changeux, 2011). Dehaene and Changeux posit “late amplification of relevant sensory activity, long-distance cortico-cortical synchronization at beta and gamma frequencies, and “ignition” of a large-scale prefronto-parietal network” as objective measurements of conscious access (Dehaene and Changeux, 2011). Neuroimaging studies on masking and binocular rivalry allow for minimal experimental differences between unconscious and conscious processing to be compared and event-related potentials and magneto-encephalography are useful for examining temporal changes on a millisecond scale (Dehaene and Changeux, 2011). Studies examining masking and binocular rivalry and even-related potentials can give us valuable insights into processes of consciousness but at this point they cannot validate or falsify any of the currently accepted consciousness models. Overall, we have proposed a dynamic global theory that is indirectly supported by basic and clinical studies; however, direct studies are needed to elucidate the detailed underlying mechanisms. Understanding how consciousness arises would greatly benefit the entire field of neuroscience and provide insights into the diagnosis and treatment of countless disorders.

**Table 1:** **Comparison of 3D default space consciousness model and other current models**.

**Questions addressed by consciousness models:**	**Integrated information theory**	**Global workspace theory**	**3D default space theory**
Does it involve a global theory involving brain and body?	Yes	Yes	Yes
Does corticothalamic activity play a major role?	Yes	Yes	Yes
Does it address underlying physiology?	No	Yes, partially	Yes
Is it an expandable model? (Can it be integrated with other models?)	Limited expandability	Limited expandability	Yes
Can the model be visualized via illustrations?	No	No	Yes
Does the model address emotions?	Yes, partially	Yes, partially	Yes
Does it address cardiorespiratory coherence?	No	No	Yes
Does it address the role of breathing on various rhythms including the brain?	No	No	Yes
Does it address the role of homeostasis and membrane potential changes?	No	No	Yes
Is the thalamus a central hub for consciousness in the model?	No	No	Yes
How is consciousness perceived?	Externally	Externally	Internally
Does the model explain the self?	No	No	Not at present
Does the model explain how of visual and other sensory inputs are integrated?	Yes	No	Yes
Can the model illustrate the etiological deficits of the following disorders:			
a) Contralateral neglect syndrome	No	No	Yes
b) Phantom limb	No	No	Yes
c) Motion sickness	No	No	Yes
d) Vertigo	No	No	Yes
e) Claustrophobia	No	No	Yes
Can the model explain anxiety vs. mind-body response and the involved underlying physiology?	No	No	Yes
Does the model address how emotions and sensory info is integrated?	No	No	Yes
Does the model have experimental evidence?	Partial	Partial	Partial
Does the model include the autonomic nervous system?	No	No	Yes
Limitations of these consciousness models:			
a) Does it identify spatiotemporal coordinates of emotions?	No	No	No
b) Does it detail the link between neural oscillations and visual and auditory consciousness?	No	No	No
c) Can the model explain the relationship of body movement to 3D default space?	N/A	N/A	No

*Green indicates that the consciousness model addresses the corresponding question while red indicates that the model does not address the question*.

**Table 2:** **Summary of 3D default space consciousness model**.

	**Layer 1**	**Layer 2**	**Layer 3**	**3D default space**
**Main function**	Forms the neural sensory memory space	Consists of limbic activity, emotions and feelings, as well as gut and viscera connections	Higher processing, consciousness, involved in filling in the sensory memory space	Involved in consciousness and homeodynamic processes
**Areas of neural activity**	Default mode network, resting state networks, reticular activating system, cardiorespiratory activity	Limbic system activity and cardiorespiratory activity	Cortical and corticothalamic activity and cardiorespiratory activity	Composed of all interconnected cells of the body and brain
**Types of oscillations**	Slow oscillations, delta^*^	Theta and beta^*^	Alpha and gamma^*^	Includes all neural oscillatory activity as well as oscillatory activity throughout the body
**Dysfunction arising from damage to layer**	Unconsciousness, coma, sleep disorders, autonomic dysfunction, etc.	Emotion disorders, amnesia, panic attacks, etc.	Contralateral neglect, cortical blindness, sensory dysfunction, etc.	All illnesses and disorders (this layer consists of all cells of the body)

* *Disclaimer: this table summarizes the main proposed characteristics of our model; however, this model requires further study to substantiate these proposals*.

### Conflict of interest statement

The authors declare that the research was conducted in the absence of any commercial or financial relationships that could be construed as a potential conflict of interest.
